# Three weeks of environmental enrichment enhance hepatic-muscular oxidative balance and decrease interleukin-6 levels in juvenile female C57BL/6 mice

**DOI:** 10.3389/fphys.2025.1626477

**Published:** 2025-08-14

**Authors:** Débora Eduarda da Silva Fidélis, Matheus Santos de Sousa Fernandes, Tiago Lacerda Ramos, Clarice Beatriz Gonçalves Silva, Allifer Rosendo Perreira, Cássia Giselle de Olivera Nóbrega, Valdênia Maria Oliveira de Souza, Fatma Hilal Yagin, Yalin Aygun, Claudia Jacques Lagranha, Mariana Pinheiro Fernandes, Reem M. Alwhaibi, Fabrício Oliveira Souto

**Affiliations:** ^1^ Keizo Asami Institute, Federal University of Pernambuco, Recife, Pernambuco, Brazil; ^2^ Postgraduate Program in Biology Applied to Health, Federal University of Pernambuco, Recife, Pernambuco, Brazil; ^3^ Laboratory of Biochemistry and Molecular Biology of Physical Exercise, Academic Center of Vitoria de Santo Antão, Federal University of Pernambuco, Vitoria de Santo Antão, Brazil; ^4^ Health Sciences Center, Department of Pharmaceutical Sciences, Federal University of Pernambuco, Recife, Pernambuco, Brazil; ^5^ Department of Biostatistics, Faculty of Medicine, Malatya Turgut Özal University, Malatya, Türkiye; ^6^ Department of Sport Management, Faculty of Sport Sciences, Inonu University, Malatya, Türkiye; ^7^ Department of Rehabilitation Sciences, College of Health and Rehabilitation Sciences, Princess Nourah bint Abdulrahman University, Riyadh, Saudi Arabia

**Keywords:** inflammation, cytokines, enzymatic activity, oxidative stress, and metabolism

## Abstract

**Introduction:**

The Environmental Enrichment (EE) promotes systemic responses through social, cognitive, sensory, and physical stimuli. However, its effects on hepatic and muscle oxidative balance, as well as on serum inflammation markers, remain unclear. Therefore, this study aimed to evaluate whether three weeks of EE could modulate hepatic and muscular oxidative balance and the inflammatory response in female C57BL/6 mice.

**Methods:**

The animals were divided into two groups: EE (*n* = 13) and Standard Environmental (SE, *n* = 11) from postnatal day 35 to 60. The EE setting included inanimate objects such as tunnels, ladders, and toys made of both wood and plastic. After three weeks, the mice were euthanized for the withdrawal of the liver, extensor digitorum longus (EDL), soleus, and blood samples.

**Results and discussion:**

EE significantly reduced body weight and malondialdehyde levels in the liver, soleus, and EDL muscles. Additionally, carbonyl levels decreased in the liver and soleus. Acute EE exposure enhanced enzymatic antioxidant activity (SOD, CAT, and GST) across all tissues, except for catalase activity in the EDL, which showed no significant difference between groups. Non-enzymatic defenses were improved, with reduced oxidized glutathione (GSSG) levels in the liver and soleus. Furthermore, EE increased the REDOX status in the liver and EDL. Sulfhydryl levels increased only in the liver. Finally, serum cytokine analysis revealed a significant reduction only in IL-6 levels. These findings suggest that three weeks of EE can modulate hepatic and muscular oxidative balance, as well as serum IL-6 levels, in juvenile female mice.

## 1 Introduction

Early studies on environmental enrichment (EE) were conducted by Donald Hebb in the 1940s and 1950s. While investigating animal behavior, Hebb observed that environmental variability could influence neurophysiological and behavioral outcomes (Muhammad et al., 2017). In the 1960s, Mark Rosenzweig and colleagues advanced this concept by demonstrating that environments enriched with social, motor, and sensory stimuli could induce significant changes in brain neuroanatomy and physiology (Diamond et al., 1964). Subsequently, during the 1990s and 2000s, Gerd Kempermann established a definitive link between EE and neurogenesis, showing that enriched environments stimulate the proliferation of neural stem cells, enhance synaptic plasticity, and modulate immunometabolic processes (Kempermann et al., 1997).

EE is a non-pharmacological approach designed to produce psychophysiological benefits at the systemic level (Fernandez et al., 2004). EE typically entails housing animals in larger enclosures that promote increased social interaction and provide access to a diverse array of objects varying in shape and texture (Bayne, 2018). These elements serve to stimulate curiosity and exploratory behavior, fostering voluntary engagement with the environment. Research has demonstrated that EE can modulate inflammatory and metabolic responses through the integrated effects of social, physical, cognitive, and sensory stimuli (Ratuski and Weary, 2022).

Metabolism encompasses a network of chemical reactions, regulated by enzymatic complexes, that maintain physiological homeostasis (Lehninger et al., 2004). Within this context, EE can induce bioenergetic adaptations by influencing lipid and glucose metabolism and enhancing mitochondrial function. Metabolic imbalance is often associated with oxidative stress (OS). EE has been shown to stimulate antioxidant pathways, increasing the expression of enzymes such as SOD and catalase (CAT), while reducing the production of pro-oxidant compounds—making it a promising strategy for OS prevention (Zhang et al., 2021). Additionally, exposure to an EE has been shown to activates the Nrf2-ARE signaling pathway, contribute to neuroprotection and improved cognitive function (Zhang et al., 2021). In addition, multiple studies have demonstrated that exposure to an EE can activate the miR-146a-5p, thereby hippocampal inflammatory response and cognitive function (Zhou et al., 2022). In aged animals, has been found that activation of Nrf2 expression mitigate this neurogenic decline and improve cognitive abilities (Ray et al., 2018). Similarly, in aging mice, has been showed that EE exposure reduced neuroinflammation and improve behavior performance under cerebral ischemia (Zhang et al., 2023). Guo et al. (2022) observed that EE reduced the damage to brain tissue caused by activation of microglia activation, decreased the level of pro-inflammatory cytokins, which may induced by microglia, protected and promote recovery to improve cognitive function after stroke (Guo et al., 2022). Grinan-Ferre et al. (2021) showed that EE induced epigenetic modifications (e.g. methylation) in the hippocampal gene expression leading to reduction in inflammation and improve in antioxidant defense, associated to NRF2 and NF-kB regulation (Griñan-Ferré et al., 2016). Lastly, Jia et al. (2023) demonstrated that in intracerebral hemorrhage, EE promoted spatial learning and memory, by changes associated with increased expression of NRF2 and BDNF. Together, these findings suggest that EE acts as a non-pharmacological intervention that activates Nrf2 signaling to counteract oxidative stress and neurodegeneration across various models (Jia et al., 2023).

Furthermore, evidence suggests that oxidative balance contributes to enhanced immune system function, aiding in the defense against pathogens and the regulation of inflammatory responses (Lauridsen, 2019a). Among the various factors involved in inflammation is the production of cytokines, including interleukin-6 (IL-6) (Lauridsen, 2019b; Kang et al., 2020). IL-6 is produced by various immune cells, including macrophages, endothelial cells, and both B and T lymphocytes (Kang et al., 2020). Under physiological conditions, IL-6 exhibits both pro-inflammatory and anti-inflammatory properties, playing a crucial role in the immune response to infections and in tissue repair (Uciechowski and Dempke, 2020). However, when its expression becomes dysregulated, IL-6 can serve as a key driver of chronic inflammation and contribute to the development of several pathological conditions, including cancer (Uciechowski and Dempke, 2020; Kumari et al., 2016). Additionally, studies suggest that various therapeutic approaches and lifestyle-related interventions can lead to a reduction in IL-6 levels, resulting in a significant decrease in OS (Robinson et al., 2020). In this context, Ferré et al. demonstrated that the application of EE significantly reduced IL-6 levels and oxidative stress markers in the hippocampus of SAMP8 mice. In the same path studies demonstrate that EE reduces chronically elevated IL-6 levels, particularly in the brain and periphery, under stress or disease conditions, thus attenuating neuroinflammation and supporting cognitive resilience. For example, EE exposure significantly decreased IL-6 levels and improve the behaviors in Alzheimer’s disease mice (Zhang et al., 2018). In a clinical study, Bruno et al. (2018) showed that EE slows, cognitive decline, especially in hypertensive individuals. This effect is accompanied by improved systemic endothelial function and preserved carotid impairment (Bruno et al., 2018). According to Singhal et al. (2014), the physical activity performed in elicits anti-inflammatory and neuromodulatory effects through interaction with several immune pathways including IL-6, reducing cognitive and memory deficits. Therefore, EE may elicit a hormetic IL-6 response—suppressing maladaptive inflammation while supporting neural plasticity—making it a promising non-pharmacological strategy in the prevention or treatment of neuropsychiatric and neurodegenerative disorders (Singhal et al., 2014). These findings suggest that EE may represent a low-cost strategy to promote neuroprotection. However, further research is needed to elucidate the effects of EE on other organs and tissues, as well as its relationship with inflammatory markers and oxidative balance.

Thus, EE acts as a systemic modulator of both metabolism and inflammation, benefiting metabolically active organs such as the liver—central to energy metabolism—and skeletal muscle, which plays a key role in nutrient uptake and utilization. These tissues are therefore important targets in immunometabolic research (Garrido et al., 2019). Therefore, this study aimed to evaluate whether 3 weeks of EE could modulate hepatic and muscular oxidative balance and the inflammatory response in female C57BL/6 mice.

## 2 Materials and methods

The procedures of the present study were conducted by the guidelines of the ethics committee on animal use at the Federal University of Pernambuco and were approved with registration number 0028/2024. All animal experiments were completed with ARRIVE guidelines and have been carried out following the United Kingdom Animal (Scientific Procedures) Act 1986 and associated guidelines, EU Directive 2010/63/EU for Animal Experiments, and the National Research Council Guide to the Care and Use of Laboratory Animals.

### 2.1 Animals and experimental design

This study was conducted using 24 female C57BL/6 mice obtained from the breeding and experimentation vivarium of the Institute Keizo Asami Institute (ILIKA/UFPE), Federal University of Pernambuco, Brazil. All animals were housed in appropriate conditions with controlled temperature (22°C ± 2°C), a 12:12 h light-dark cycle, and had access to wood shavings, as well as water and commercial feed *ad libitum* (Nuvilab CR-1, Quimtias, Brazil). At 35 days of age, after weaning, an independent and blinded researcher (MSSF) randomly allocated the mice to their respective experimental groups: standard environment (SE, n = 11) or environmental enrichment (EE, n = 13). This type of randomization aimed to significantly reduce sample selection bias. At 60 days of age, animals were euthanized, and liver, skeletal muscles (soleus and extensor digitorum longus), and whole blood were collected for analysis.

### 2.2 Environmental enrichment protocol

**FIGURE 1:**
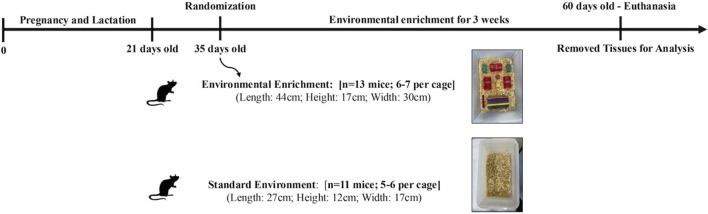
Experimental design of a 3-week Environmental Enrichment intervention in juvenile female C57BL/6 mice.

The environmental enrichment (EE) protocol was adapted to provide sensory, cognitive, and motor stimulation using inanimate objects, aiming to establish a greater capacity for stimulation and socialization (Zhang et al., 2023). Cages for the EE group were larger (44 × 17 × 30 cm) and housed six to seven animals, while standard cages (SE group) measured 27 × 12 × 17 cm and housed three to four animals. Enrichment objects included plastic and wooden tunnels, ladders, dens, and toys. The objects were changed weekly to promote diversity and increase the complexity of the environment, thus promoting a higher level of stimulation, the objects were changed weekly. The intervention lasted for 3 weeks. Both groups had free access to food, water, and bedding materials (Kempermann, 2019). The overall experimental timeline is illustrated in Figure 1.

### 2.3 Euthanasia and tissue removal

At 60 days of age and the end of the third week of environmental enrichment, the animals were euthanized. Euthanasia was performed via intraperitoneal injection of a combination of ketamine hydrochloride (0.1 mL/kg) and xylazine (10 mg/kg). Following exsanguination, the liver, soleus, and extensor digitorum longus muscles were collected. The harvested biological tissues were then stored in a freezer at −80°C.

### 2.4 Body weight measurement

To assess the metabolic responses elicited by the environmental enrichment (EE) protocol, the animals were weighed weekly using a standardized scale with a precision of 0.01 kg (Yamato model, Japan).

### 2.5 Bradford assay for protein content

The protein concentration of the liver and skeletal muscle suspension was determined by the Bradford method (He, 2011). This complex absorbs at a wavelength of 595 nm. The absorbance is directly proportional to the protein concentration in the solution analyzed, where a 1% bovine serum albumin solution will be used as a standard.

### 2.6 Biomarkers of oxidative stress

#### 2.6.1 Malonaldehyde (MDA)

The colorimetric technique standardized by Buege and Aust (1978) was used to measure MDA to evaluate lipid peroxidation. For this purpose, an aliquot of the liver and soleus muscle homogenate was associated with 30% trichloroacetic acid (TCA) and Tris-HCl (3mM, pH 7.4). After this moment, centrifugation was performed at 3.000 revolutions per minute (RPM) for 10 min, and 0.73% Thiobarbituric acid was added, reacting with the lipid peroxidation products and forming a pink-colored compound. Then, the compound was incubated for 15 minutes at 1000°C. After this process, a glass cuvette was used to read the absorbance at 535 nm. The results were expressed in millimoles per milligram (mM/mg) of protein (Buege and Aust, 1978).

#### 2.6.2 Protein oxidation analysis

To assess the level of protein oxidation, the carbonyl content was determined, as initially described by Reznick and Packer (1994). TCA 30% was added to the homogenate, kept on ice, and centrifuged for 14 min at 1.180 g. After centrifugation, the supernatant was discarded, and the pellet was resuspended in 10 mM 2,4-dinitrophenylhydrazine (DNPH) and incubated in a dark room at room temperature for 1 hour with agitation programmed every 15 min. After an incubation period of 28 h, the samples were washed in ethyl/acetate buffer and centrifuged three times. The pellet was resuspended in 6 M guanidine hydrochloride, incubated for 30 min at 37°C, and the absorbance was measured at 370 nm. The results were expressed in mM/mg of protein (Reznick and Packer, 1994).

### 2.7 Antioxidant enzymatic activity

#### 2.7.1 Superoxide dismutase (SOD)

Superoxide dismutase activity was assessed using the adrenaline oxidation method, which is the responsibility of SOD. This phenomenon can be measured in a spectrophotometer at 480 nm. In a 1 mL quartz cuvette, 0.1M carbonate buffer (pH 10.2), 0.1 mM EDTA, sample, and 150 mM adrenaline were added. The decrease in absorbance was monitored for 90 s at 30°C at a wavelength of 480nm, and the results were expressed in millimoles per minute per milligram (mM/min/mg) of protein (Misra and Fridovich, 1972).

#### 2.7.2 Catalase assay (CAT)

The activity of this enzyme was evaluated through the decomposition of hydrogen peroxide (H_2_O_2_), verified by an absorbance of 240 nm at a temperature of 20°C. The procedure consisted of adding the sample to a reaction medium containing 50 mM phosphate buffer (pH 7.0), followed by the addition of H_2_O_2_ (0.3 mM). The absorbance was analyzed for 3 min, and the results were expressed in mM/min/mg of protein (Hugo and Lester, 1984).

#### 2.7.3 Glutathione S-transferase (GST)

The activity of Glutathione-S-Transferase (GST), described by Habig (1974), was performed. The procedure included adding 0.1M potassium phosphate buffer (pH 6.5), 1 mM EDTA, 1 mM GSH, the sample, and 1 mM 1-chloro-2,4-dinitrobenzene (CDNB). The enzymatic activity was evaluated from the formation of 2,4-dinitrophenyl-s-glutathione (DNP-SG) per minute at 30°C, monitored by a spectrophotometer with a wavelength equal to 340 nm. The GST activity was expressed in mM/min/mg of protein (Habig et al., 1974).

### 2.8 Non-enzymatic antioxidant system

Reduced glutathione was assayed as previously described by Hissin and Hilf. To assess GSH levels, the samples were diluted tenfold in 0.1 M phosphate buffer containing 5 mM EDTA (pH 8.0). After this, the sample was incubated with 1 mg/mL o-phthaldialdehyde (OPT) at room temperature for 15 min, and after that, fluorescence was evaluated at 350 nm excitation and 420 nm emission. The oxidized glutathione (GSSG) levels were evaluated by incubation of samples with 40 mM N-ethylmaleimide for 30 min at RT, followed by the addition of 100 mM NaOH buffer, the result expressed as µM/mg of protein. The ratio of GSH/GSSH27 determined the REDOX state (Hissin and Hilf, 1976).

#### 2.8.1 Sulfhydryl’s

The total and sulfhydryl group content of protein was described by Aksenov and Markesbery (2001). The reduction of 5,5-dithiobis (2-nitrobenzoic acid) by thiol groups was measured in a homogenate of 200 mg of protein, resulting in the generation of a yellow pigment-like compound, the absorption of which was measured spectrophotometrically at 412 nm (Aksenov and Markesbery, 2001). The results were expressed in mM/mg of protein.

### 2.9 Cytokine assay in serum

Cytokine levels were quantified using the BD™ Cytometric Bead Array (CBA) (BD Biosciences, USA) to detect Interleukin-2 (IL-2), Interleukin −4 (IL-4), Interleukin −6 (IL-6), Interleukin-10 (IL-10), Interleukin 17A (IL -17A), Interferon gamma (IFN-γ), Tumor necrosis factor alpha (TNF-α). Aliquots of the serum samples were thawed and diluted with assay diluent (1:2 v/v), and CBA analysis was performed according to the manufacturer’s protocol (BD Pharmingen™). The reading was performed using a BD Accuri™ C6 flow cytometer. The analysis was performed using FCAP Array software (BD Biosciences, USA). The results were expressed in picograms per milliliter (pg/mL) (Filgueira et al., 2023).

### 2.10 Statistical analysis

Initially, we performed a descriptive analysis of the data associated with the Shapiro-Wilk normality test. Then, the data were expressed as mean and standard deviation. To compare the groups of normal data, the unpaired Student’s t-test with Welch’s correction was used. However, for data that did not present a normal distribution, we used the Mann-Whitney test. The effect size (Hedges’ g) was calculated by comparing the means and standard deviations of the post-intervention phase. The classification used was as follows: ignored (0.0 to <0.2), small (≥0.2 to <0.5), moderate (≥0.5 to <0.8), large (≥0.8 to <1.3), and very large (≥1.3) (Cohen, 1988). Significance was set at p < 0.05 (5%). GraphPad Prism version 10 software (GraphPad Software Inc., La Jolla, CA, USA) was used for data analysis.

## 3 Results

### 3.1 Effects of environmental enrichment on body weight evolution

**FIGURE 2:**
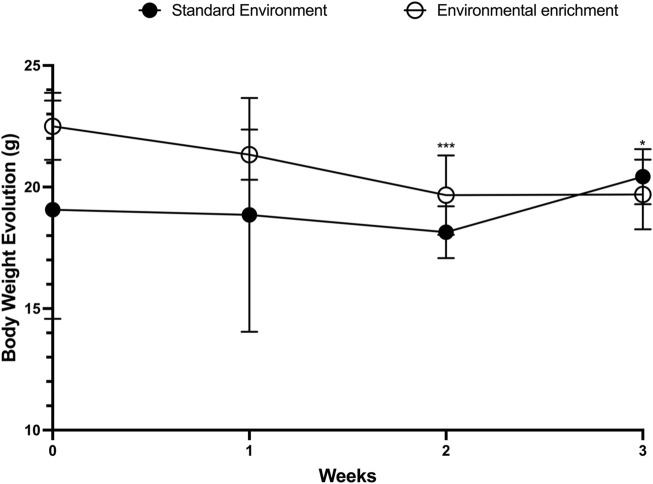
Comparison of body weight evolution in juvenile female C57BL/6 mice during 3 weeks of Environmental Enrichment. Data expressed as mean ± standard deviation. *p < 0.05; ***p < 0.001; n = 7–13 per group. Differences were assessed by unpaired Student’s t-test. EE, Environmental Enrichment (EE); Standard Environment (SE).

We initially evaluated the effects of EE on the weekly average body weight of female mice. To ensure baseline homogeneity between groups, pre-intervention body weights were compared and found to be statistically similar (SE: 16.23 ± 5.30 g vs EE: 16.62 ± 5.81 g; p = 0.86). Likewise, no significant difference was observed after the first week of EE exposure (SE: 18.00 ± 3.97 g vs EE: 19.23 ± 2.52 g; p = 0.38). However, by the end of the second week, animals in the EE group exhibited a significant increase in body weight compared to the SE group (SE: 18.36 ± 1.12 g vs EE: 23.38 ± 3.38 g; p = 0.0005; Δ = +27.34%). Interestingly, by the end of the third week, a significant reduction in body weight was observed in the EE group relative to the SE group (SE: 20.67 ± 1.03 g vs EE: 19.36 ± 1.28 g; p = 0.04; Δ = −6.33%) (Figure 2).

### 3.2 Markers of hepatic and muscular oxidative stress

**FIGURE 3:**
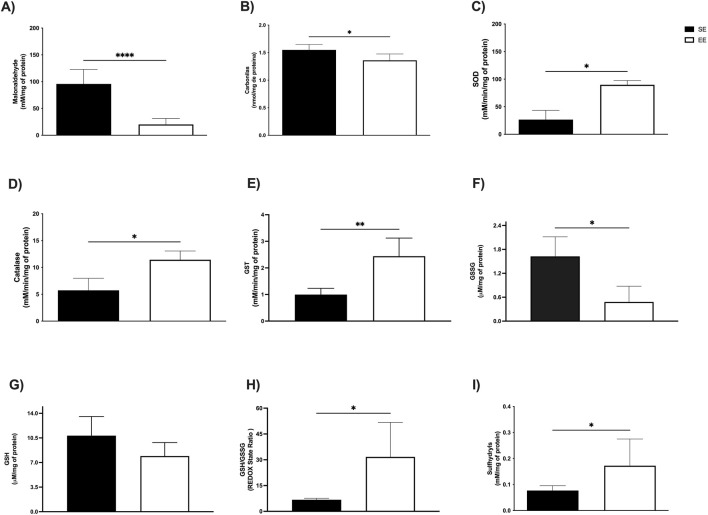
Hepatic oxidative balance of juvenile female C57BL/6 mice after 3 weeks of Environmental Enrichment. **(A)** Malonaldehyde; **(B)** Carbonyls; **(C)** Superoxide Dismutase (SOD); **(D)** Catalase; **(E)** Glutathione S Transferase (GST); **(F)** Oxidized Glutathione (GSSG); **(G)** Reduced Glutathione (GSH); **(H)** REDOX State (GSH/GSSG ratio); **(I)** Sulfhydryl’s. Data expressed as mean ± standard deviation. *p < 0.05; **p < 0.01; ****p < 0.0001; n = three to seven per group. Differences assessed using the unpaired Student’s t-test or the Mann Whitney test, according to data normality. EE, Environmental Enrichment (EE); Standard Environment (SE).

We next investigated the effects of 3 weeks of EE on oxidative stress biomarkers in the liver and skeletal muscle. In hepatic tissue, levels of MDA—a key marker of lipid peroxidation and cellular damage—were significantly reduced in the EE group compared to the SE group (SE: 95.77 ± 27.08 mM/mg protein vs EE: 20.19 ± 11.14 mM/mg protein; p < 0.0001; Δ = −78.91%) (Figure 3A). Additionally, protein carbonyl content, an indicator of protein oxidation, was also significantly decreased following EE (SE: 1.55 ± 0.09 mM/mg protein vs EE: 1.36 ± 0.11 mM/mg protein; p = 0.04; Δ = −12.25%) (Figure 3B).

**FIGURE 4:**
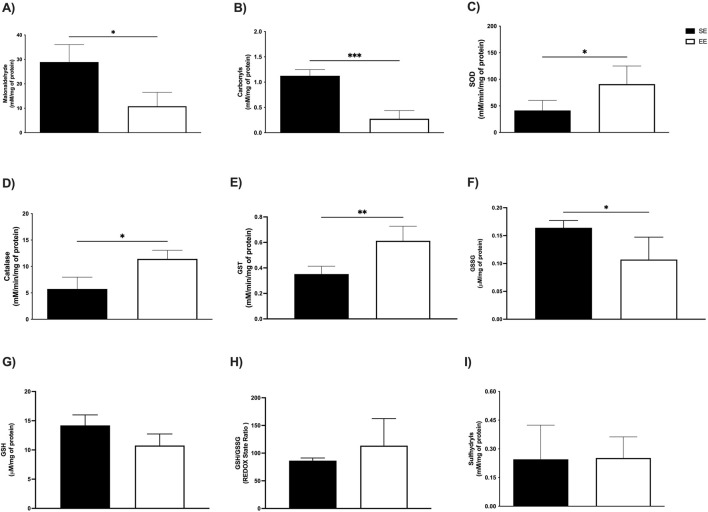
Assessment of levels of oxidative stress markers; **(A)** Malonaldehyde; **(B)** Carbonyls; Antioxidant enzymatic activity **(C)** Superoxide Dismutase (SOD); **(D)** Catalase; **(E)** Glutathione S Transferase (GST); and Non-enzymatic antioxidant compounds; **(F)** Oxidized Glutathione (GSSG); **(G)** Reduced Glutathione (GSH); **(H)** REDOX State (GSH/GSSG ratio); **(I)** Sulfhydryl’s in the soleus of Juvenile Female C57BL/6 Mice after 3 weeks of Environmental Enrichment. Data expressed as mean ± standard deviation. *p < 0.05; **p < 0.01; ***p < 0.001; n = = three to seven per group. Differences assessed using the unpaired Student’s t-test or the Mann Whitney test, according to data normality. EE, Environmental Enrichment (EE); Standard Environment (SE).

**FIGURE 5:**
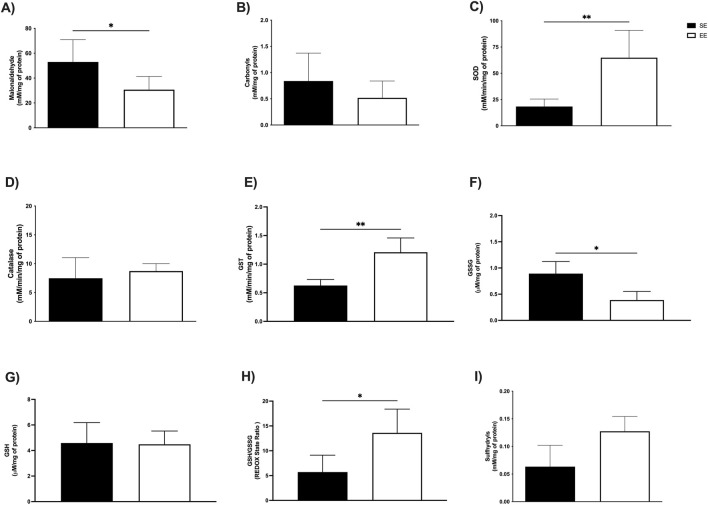
Effects of 3 weeks of environmental enrichment on markers of oxidative stress and enzymatic and non-enzymatic antioxidant defenses in the extensor digitorum longus in juvenile female C57BL/6 mice. **(A)** Malonaldehyde; **(B)** Carbonyls; **(C)** Superoxide Dismutase (SOD); **(D)** Catalase; **(E)** Glutathione S Transferase (GST); **(F)** Oxidized Glutathione (GSSG); **(G)** Reduced Glutathione (GSH); **(H)** REDOX State (GSH/GSSG ratio); **(I)** Sulfhydryl’s *p < 0.05; **p < 0.01; n = = three to seven per group. Data expressed as mean ± standard deviation. Differences assessed using the unpaired Student’s t-test or the Mann Whitney test. EE, Environmental Enrichment (EE); Standard Environment (SE).

In the soleus muscle, EE led to a significant reduction in MDA levels (SE: 28.91 ± 7.16 mM/mg protein vs EE: 10.81 ± 5.70 mM/mg protein; p = 0.02; Δ = −62.60%) (Figure 4A), as well as in protein carbonyl content (SE: 1.12 ± 0.12 mM/mg protein vs EE: 0.81 ± 0.27 mM/mg protein; p = 0.0002; Δ = −27.67%) (Figure 4B). Similarly, in the extensor digitorum longus muscle, EE significantly decreased MDA levels compared to the SE group (SE: 52.99 ± 17.87 mM/mg protein vs EE: 30.67 ± 10.59 mM/mg protein; p = 0.02; Δ = −42.12%) (Figure 5A). However, no significant differences were observed in protein carbonyl content between groups (SE: 0.83 ± 0.53 mM/mg protein vs EE: 0.51 ± 0.32 mM/mg protein; p = 0.41) (Figure 5B).

### 3.3 Antioxidant enzyme activity

To assess the effects of EE on the antioxidant enzyme system, we measured the activities of superoxide dismutase (SOD), catalase, and glutathione S-transferase (GST) in both the liver and soleus muscle. In the liver, SOD activity was significantly elevated following 3 weeks of EE compared to the standard environment (SE) group (SE: 26.67 ± 16.80 mM/min/mg protein vs EE: 89.83 ± 7.67 mM/min/mg protein; p = 0.01; Δ = +236.82%) (Figure 3C). Similarly, catalase activity increased significantly (SE: 6.98 ± 0.49 mM/min/mg protein vs EE: 10.58 ± 1.30 mM/min/mg protein; p = 0.0006; Δ = +53.00%) (Figure 3D), as did GST activity (SE: 0.99 ± 0.23 mM/min/mg protein vs EE: 2.61 ± 0.59 mM/min/mg protein; p = 0.002; Δ = +163.63%) (Figure 3E).

In the soleus muscle, we observed comparable enhancements in enzymatic activity. SOD levels were significantly higher in the EE group (SE: 41.83 ± 18.88 mM/min/mg protein vs EE: 91.00 ± 33.98 mM/min/mg protein; p = 0.02; Δ = +117.54%) (Figure 4C), along with catalase (SE: 5.73 ± 2.23 mM/min/mg protein vs EE: 11.44 ± 1.62 mM/min/mg protein; p = 0.02; Δ = +99.65%) (Figure 4D), and GST (SE: 0.35 ± 0.06 mM/min/mg protein vs EE: 0.61 ± 0.11 mM/min/mg protein; p = 0.002; Δ = +74.28%) (Figure 4E). In the extensor digitorum longus, EE also led to a significant increase in SOD activity (SE: 18.33 ± 7.09 mM/min/mg protein vs EE: 64.86 ± 26.04 mM/min/mg protein; p = 0.002; Δ = +253.84%) (Figure 5C) and GST activity (SE: 0.62 ± 0.10 mM/min/mg protein vs EE: 1.20 ± 0.24 mM/min/mg protein; p = 0.001; Δ = +93.54%) (Figure 5E). No significant difference was observed in catalase activity between groups in this tissue (SE: 7.47 ± 3.57 mM/min/mg protein vs EE: 8.71 ± 1.29 mM/min/mg protein; p = 0.61) (Figure 5D).

### 3.4 Non-enzymatic antioxidant defenses

We also examined non-enzymatic antioxidant markers following 3 weeks of EE, including oxidized (GSSG) and reduced (GSH) glutathione, redox status, and sulfhydryl content. In the liver, EE significantly reduced GSSG levels compared to the SE group (SE: 16.27 ± 0.48 μM/mg protein vs EE: 0.48 ± 0.39 μM/mg protein; p = 0.03; Δ = −97.05%) (Figure 3F). No significant changes were observed in GSH levels (SE: 10.82 ± 2.72 μM/mg protein vs EE: 7.92 ± 1.92 μM/mg protein; p = 0.19) (Figure 3G). However, EE led to a marked increase in redox status (SE: 6.72 ± 0.83 μM/mg protein vs EE: 30.13 ± 14.62 μM/mg protein; p = 0.04; Δ = +348.36%) (Figure 3H), and sulfhydryl content (SE: 0.07 ± 0.01 mM/mg protein vs EE: 0.17 ± 0.10 mM/mg protein; p = 0.01; Δ = +142.85%) (Figure 3I).

In the soleus muscle, EE significantly reduced GSSG levels (SE: 0.16 ± 0.01 μM/mg protein vs EE: 0.10 ± 0.04 μM/mg protein; p = 0.01; Δ = −37.50%) (Figure 4F). No significant differences were observed in GSH levels (SE: 14.20 ± 1.79 μM/mg protein vs EE: 10.76 ± 1.96 μM/mg protein; p = 0.05) (Figure 4G), redox status (SE: 86.40 ± 4.83 U/A vs EE: 113.5 ± 48.86 U/A; p = 0.23) (Figure 4H), or sulfhydryl content (SE: 0.24 ± 0.17 mM/mg protein vs EE: 0.27 ± 0.09 mM/mg protein; p = 0.62) (Figure 4I).

Finally, in the extensor digitorum longus, EE significantly reduced GSSG levels (SE: 0.89 ± 0.23 μM/mg protein vs EE: 0.38 ± 0.16 μM/mg protein; p = 0.04; Δ = −57.30%) (Figure 5F). GSH levels remained unchanged (SE: 4.58 ± 1.59 μM/mg protein vs EE: 4.48 ± 1.03 μM/mg protein; p = 0.92) (Figure 5G). However, redox status was significantly improved in the EE group (SE: 5.70 ± 3.41 U/A vs EE: 13.60 ± 4.78 U/A; p = 0.03; Δ = +137.59%) (Figure 5H). No significant difference was found in sulfhydryl levels (SE: 0.06 ± 3.41 mM/mg protein vs EE: 0.12 ± 4.78 mM/mg protein; p = 0.08) (Figure 5I).

### 3.5 Cytokine levels

**FIGURE 6:**
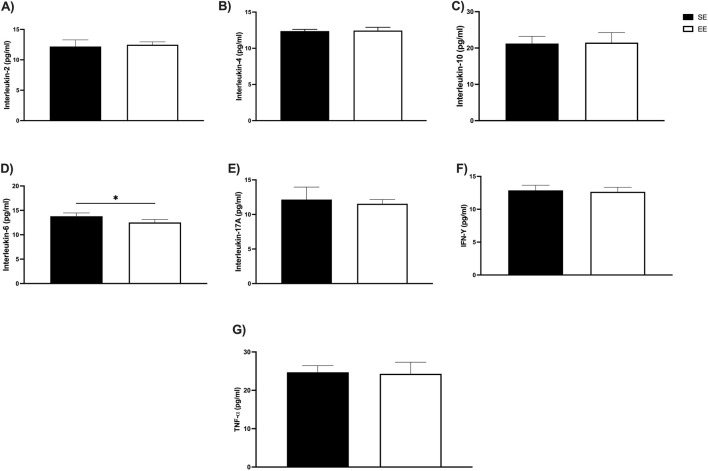
Impacts of 3 weeks of Environmental Enrichment on serum cytokine levels linked to the Th1/Th2 profile in juvenile female C57BL/6 mice; **(A)** Interleukin-2 (IL-2); **(B)** Interleukin-4 (IL-4); **(C)** Interleukin-10 (IL-10); **(D)** Interleukin-6 (IL-6); **(E)** Interleukin-17A (IL-17A); **(F)** Interferon gamma (IFN-γ); **(G)** Tumor Necrosis Factor Alpha (TNF-α) in the Juvenile Female C57BL/6 Mice after 3 weeks of Environmental Enrichment; *p < 0.05; n = 5 per group. Data expressed as mean ± standard deviation. Differences assessed using the unpaired Student’s t-test. EE, Environmental Enrichment (EE); Standard Environment (SE).

Finally, we assessed serum levels of anti- and pro-inflammatory cytokines associated with the Th1/Th2 immune response after 3 weeks of EE. Among the anti-inflammatory cytokines, no significant differences were observed in IL-2 levels (SE: 12.20 ± 1.08 pg/mL vs EE: 12.48 ± 0.47 pg/mL; p = 0.60) (Figure 6A), IL-4 (SE: 12.37 ± 0.22 pg/mL vs EE: 12.45 ± 0.44 pg/mL; p = 0.74) (Figure 6B), or IL-10 (SE: 21.37 ± 1.99 pg/mL vs EE: 21.49 ± 2.77 pg/mL; p = 0.87) (Figure 6C). In contrast, regarding pro-inflammatory cytokines, EE significantly reduced IL-6 levels compared to the SE group (SE: 13.79 ± 0.68 pg/mL vs EE: 12.53 ± 0.58 pg/mL; p = 0.03; Δ = −9.13%) (Figure 6D). No significant changes were observed in IL-17A (SE: 12.16 ± 1.79 pg/mL vs EE: 11.55 ± 0.60 pg/mL; p = 0.50) (Figure 6E), IFN-γ (SE: 12.88 ± 0.77 pg/mL vs EE: 12.65 ± 0.69 pg/mL; p = 0.63) (Figure 6F), or TNF-α levels (SE: 24.71 ± 1.72 pg/mL vs EE: 24.29 ± 3.01 pg/mL; p = 0.79) (Figure 6G) after the EE intervention.

### 3.6 Effect size

**FIGURE 7:**
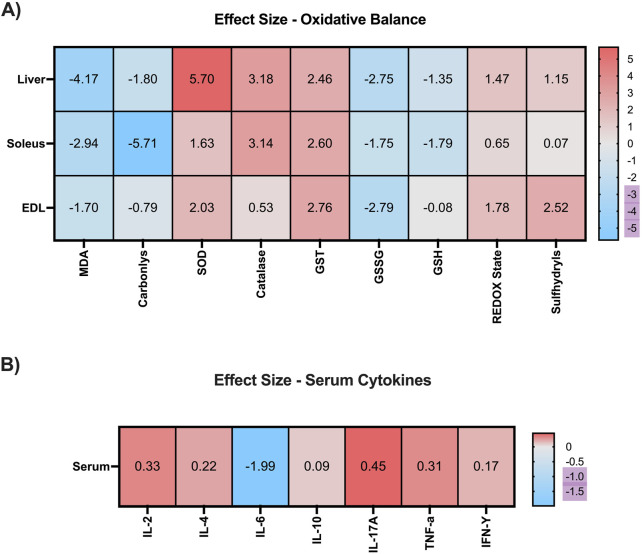
Heat map with effect size after 3 weeks of Environmental Enrichment in juvenile female C57BL/6 mice. **(A)** Oxidative Balance Markers (Malonaldehyde; Carbonyls; Superoxide Dismutase (SOD); Catalase; Glutathione S Transferase (GST); Oxidized Glutathione (GSSG); Reduced Glutathione (GSH); REDOX State (GSH/GSSG ratio); Sulfhydryl’s in the liver, soleus and extensor digitorum longus muscles. **(B)** Serum Cytokines (Interleukin-2 (IL-2); Interleukin-4; Interleukin-10; Interleukin-6; Interleukin-17A; Interferon gamma (IFN-γ); Tumor Necrosis Factor Alpha (TNF-α). Ignored (0.0 to <0.2), small (≥0.2 to <0.5), moderate (≥0.5 to <0.8), large (≥0.8 to <1.3), and very large (≥1.3).

To quantify the magnitude of the effects induced by EE on hepatic and muscular oxidative balance markers, as well as on serum cytokine levels, we employed Hedges’ g as a measure of effect size (Figure 7A). In hepatic tissue, EE produced a very large reduction in malondialdehyde (MDA) and protein carbonyl levels (g = −4.17 and g = −1.80, respectively). Additionally, EE induced very large increases in the activity of antioxidant enzymes, including superoxide dismutase (SOD; g = 5.70), catalase (g = 3.18), and glutathione S-transferase (GST; g = 2.46). Regarding non-enzymatic antioxidant defenses, EE promoted very large reductions in GSSG (g = −2.75) and GSH (g = −1.35) levels, alongside very large increases in redox status (g = 1.47) and sulfhydryl content (g = 1.15).

In the soleus muscle, EE also elicited very large reductions in MDA (g = −2.94) and carbonyl levels (g = −5.71). For enzymatic antioxidants, EE induced very large effects on SOD (g = 1.63), catalase (g = 3.14), and GST (g = 2.60) activity. Within the non-enzymatic antioxidant system, EE produced very large reductions in GSSG (g = −1.75) and GSH (g = −1.79), a moderate increase in redox status (g = 0.65), and a negligible effect on sulfhydryl levels (g = 0.07).

In the extensor digitorum longus, EE resulted in a very large reduction in MDA levels (g = −1.70) and a moderate reduction in carbonyl content (g = −0.79). Regarding enzymatic antioxidant activity, very large increases were observed in SOD (g = 2.03) and GST (g = 2.76), while catalase showed a moderate increase (g = 0.53). For non-enzymatic defenses, EE promoted a very large reduction in GSSG (g = −2.79), a negligible effect on GSH (g = −0.08), and very large increases in both redox status (g = 1.78) and sulfhydryl levels (g = 2.52).

Regarding serum cytokines, 3 weeks of EE produced small increases in IL-2 (g = 0.33), IL-4 (g = 0.22), IL-17A (g = 0.45), and TNF-α (g = 0.31), as well as a negligible effect on IFN-γ (g = 0.17). However, a very large reduction was observed in IL-6 levels (g = −1.99), suggesting a marked anti-inflammatory effect induced by EE (Figure 7B).

## 4 Discussion

The present study investigated the impact of a 3-week EE protocol on hepatic and skeletal muscle oxidative balance, as well as on systemic inflammatory responses, in juvenile female mice. Our results demonstrate that EE significantly reduced body weight and oxidative stress markers, while concurrently enhancing enzymatic antioxidant defenses in both the liver and skeletal muscle tissues. Furthermore, EE led to a notable increase in non-enzymatic antioxidant components, particularly in the liver and the extensor digitorum longus muscle. With respect to systemic inflammation, the EE intervention was associated with a significant reduction in circulating IL-6 levels after 3 weeks.

Scientific literature indicates that body weight is an important indicator of metabolic health in both humans and animals. Variations in this parameter may reflect conditions such as malnutrition or obesity, both of which can lead to deleterious effects, including inflammation, oxidative stress (OS), and mitochondrial dysfunction. The significant reduction in body weight observed in juvenile female mice after 3 weeks of EE may be associated with increased voluntary physical activity, primarily of an aerobic nature, resulting from the animals’ continuous environmental exploration under EE conditions. Aerobic activities are characterized by the recruitment of a higher number of fatty acids, whose entry into the mitochondria is facilitated by the enzyme carnitine palmitoyltransferase one alpha (CPT-1α) (Schlaepfer and Joshi, 2020; Cho et al., 2014). This promotes the beta-oxidation process, leading to a significant production of adenosine triphosphate (ATP). Studies in both humans and animal models indicate that aerobic physical activity and exercise effectively contribute to body weight regulation under both healthy and pathological conditions (Fernandes et al., 2020; Oppert et al., 2023). Furthermore, aerobic activity is known to positively influence hormonal regulation in women. In this sense, studies in animal models have shown that aerobic physical activity promoted by EE can reduce hormonal fluctuations, particularly in the secretion of estrogen and progesterone (Gresack et al., 2007; De la Cruz Borthiry et al., 2025; Sager et al., 2018). This hormonal stabilization is associated with improved body weight and fat distribution, as well as a reduction in adverse effects typically observed during the menstrual cycle and brings benefits to female health.

Under physiological conditions, communication between the liver and muscle is essential for energy homeostasis. This interaction involves the oxidation of carbohydrates and lipids within these tissues, aimed at increasing the bioavailability of ATP during periods of high energy demand, such as physical activity and exercise (Moghetti et al., 2016; Atayik et al., 2024). Conversely, inefficiencies in this process can result in OS, which is associated with chronic diseases, such as cardiovascular diseases and cancer (Senoner and Dichtl, 2019; Klaunig, 2018). The reduction in OS markers may be primarily associated with the downregulation of the hypothalamic-pituitary-adrenal (HPA) axis following EE. It is well established that exposure to stressful environments stimulates the secretion of corticotropin-releasing hormone (CRH) by the hypothalamus. CRH acts on the anterior pituitary to increase the release of adrenocorticotropic hormone (ACTH), which in turn stimulates the adrenal cortex to produce glucocorticoids, such as corticosterone in rodents (Lightman et al., 2002; Kokras et al., 2021). Elevated levels of these hormones are known to promote the overproduction of reactive oxygen species (ROS) and reactive nitrogen species (RNS), which, associated with the reduction of antioxidant defense systems, promote the OS (Spiers et al., 2014). Conversely, evidence suggests that EE can attenuate HPA axis hyperactivity, resulting in decreased glucocorticoid levels. This modulation contributes to enhanced stress resilience and protects the cellular environment from damage caused by lipid peroxidation and protein oxidation, thereby exerting a positive impact on liver and muscle function (Spiers et al., 2014; de Sousa Fernandes et al., 2024; Smail et al., 2020; Wiedmer et al., 2012; Braun et al., 2011).

To ensure cellular homeostasis, antioxidant defense systems must function effectively. Our results revealed a significant enhancement in enzymatic antioxidant activity in the liver and the extensor digitorum longus muscle after EE. These antioxidant enzymes constitute a crucial part of the first line of defense against OS and act synergistically to neutralize ROS, mainly. Superoxide dismutase (SOD) catalyzes the conversion of the superoxide radical (O_2_•^-^) into hydrogen peroxide (H_2_O_2_). Although H_2_O_2_ is less reactive than superoxide, it can still give rise to highly damaging hydroxyl radicals (•OH) through the Fenton reaction. Catalase subsequently detoxifies H_2_O_2_, converting it into water and molecular oxygen, thereby preventing further oxidative damage. Among lifestyle-related interventions, EE was able to potentiate the activity of these antioxidant enzymes in several conditions, including exposure to toxic agents and disease states (Mármol et al., 2015; Ramos et al., 2024).

In addition, Powers et al. (2022) demonstrated that increased voluntary locomotor activity, as promoted by enrichment protocols, contributes to the activation of the nuclear factor erythroid 2-related factor 2 (NRF2) signaling pathway. This transcription factor plays a key role in regulating the expression of genes involved in cellular antioxidant defense (Powers and Jackson, 2008; Powers et al., 2022). In the context of our findings, the activation of this factor in the liver and skeletal muscle appears to be associated with the upregulation of key antioxidant enzymes, such as SOD and catalase. This response likely contributes to the maintenance of mitochondrial homeostasis and plays a crucial role in mitigating oxidative stress, thereby supporting tissue function and metabolic balance (Oppert et al., 2023; Gresack et al., 2007).

Furthermore, regarding non-enzymatic antioxidant defense, it was observed in the liver that EE promotes improvements in GSSG/GSH levels, which are important modulators of genes associated with mitochondrial biogenesis and lipid metabolism, promoting improvements in the antioxidant response (Powers and Jackson, 2008; Powers et al., 2022). We then assessed sulfhydryl levels and REDOX status in the liver and skeletal muscle after 3 weeks of intervention. EE is an important tool in modulating oxidative balance and sulfhydryl levels in both tissues of these mice, enhancing positive effects on the antioxidant response. In the liver, EE can improve REDOX status by increasing glutathione availability and influencing the sulfhydryl system. This contributes to an environment less conducive to oxidative damage. This effect can be observed both by increased antioxidant enzyme response and by improved overall REDOX balance (Bernardo et al., 2024). In addition, this non-pharmacological tool promotes muscle homeostasis by optimizing the REDOX balance. Zuo and Pannell (2015) indicate that skeletal muscle benefits from this modulation of oxidative balance, which in turn promotes greater muscle endurance and recovery after oxidative damage. Our findings after 3 weeks were similar, showing an increase in sulfhydryl levels and overall REDOX state in the liver and extensor digitorum longus, suggesting that the cell is in a healthy reducing environment (Zuo and Pannell 2015).

We then evaluated several cytokines linked to the activity of T helper lymphocytes one and 2 (Th1/Th2), which are related to the body’s defense against pathogens, including viruses and bacteria. The results showed that 3 weeks of EE were able to significantly reduce the levels of IL-6 only. This cytokine is produced rapidly and transiently, according to stimuli resulting from infections and tissue injuries, contributing to the host defense system, hematopoiesis, and immunological reactions (Tanaka et al., 2014). Corroborating our findings, Xu et al. demonstrated a significant decrease in IL-6 assessed by enzyme-linked immunosorbent assay (ELISA) under chronic unpredictable mild stress conditions after 3 weeks of EE (Xu et al., 2022). Several studies have shown that it is essential to prevent dysfunctions in the production of this cytokine, which is essential for preventing chronic inflammation, metabolic pathologies, including cardiovascular and obesity, as well as autoimmune diseases. However, IL-6 under physiological conditions and depending on the biological tissue that produces it has several important functions for maintaining health, through the regulation of energetic and glycemic homeostasis (Pedersen and Febbraio, 2008; Borer et al., 2023).

These processes occur due to the production of IL-6 by skeletal muscle cells stimulated in response to physical exercise protocols, mainly. One of the explanations for such benefits is the anti-inflammatory activity of this cytokine, which, once produced in the muscle microenvironment, acts to inhibit the production of pro-inflammatory factors including tumor necrosis factor alpha (TNF-α), which is related to numerous pathological conditions such as insulin resistance and oxidative stress, which in turn compose the genesis of chronic degenerative diseases including diabetes mellitus and cancer (Kau et al., 2020; Yao et al., 2014). In addition to its immunomodulatory function, IL-6 is closely associated with oxidative stress in a bidirectional relationship: reactive oxygen species (ROS) can upregulate IL-6 expression via redox-sensitive pathways such as NF-κB and AP-1 (Mittal et al., 2014; Reuter et al., 2010), while IL-6 can further amplify oxidative stress by activating pro-inflammatory signaling cascades and recruiting ROS-generating immune cells (Tanaka et al., 2014; Scheller et al., 2011). This creates a positive feedback loop that contributes to chronic inflammation and tissue damage. Interestingly, EE—which provides physical, sensory, and cognitive stimulation—has been shown to modulate both IL-6 levels and oxidative stress. EE enhances antioxidant defenses by upregulating enzymes such as superoxide dismutase (SOD) and catalase, while reducing ROS production, lipid peroxidation, and oxidative DNA damage (Kobilo et al., 2011; Zhang et al., 2017). At the same time, EE lowers IL-6 and other pro-inflammatory cytokines, possibly through NF-κB inhibition and activation of Nrf2-mediated antioxidant pathways (Jha et al., 2011; Rojas et al., 2009), thereby promoting an anti-inflammatory state. Despite growing interest, few studies have specifically investigated the effects of enriched environments (particularly acute exposure) on serum and tissue IL-6 levels under physiological conditions. Addressing this gap is crucial for deepening our understanding of IL-6’s regulatory mechanisms and for exploring the potential of enriched environments as a non-pharmacological strategy to prevent or mitigate a range of clinical and pathological conditions.

## 5 Strengths, limitations, and future directions

To the best of our knowledge, this is the first study in the scientific literature to examine the effects of a 3-week EE protocol on hepatic and muscular oxidative balance in juvenile female mice. These tissues play a central role in the regulation of numerous physiological processes essential for maintaining health and survival, including energy metabolism through the oxidation and storage of macronutrients, particularly carbohydrates. For future research, we recommend investigating genes associated with glucose metabolism, as well as their potential relationship with oxidative balance outcomes, to provide a more comprehensive understanding of these mechanisms. Furthermore, this study also represents a pioneering effort in exploring the potential effects of EE on the cytokine profile associated with Th1/Th2 lymphocyte responses.

Comprehensive data on the profile and viability of immune cells—such as monocytes, leukocytes, and lymphocytes—would significantly enhance the integrative understanding of the biological systems and processes influenced by EE. The inclusion of juvenile female mice in association with EE in this study is particularly noteworthy, given that most existing research has predominantly utilized male subjects, primarily to circumvent potential confounding effects associated with hormonal fluctuations during the estrous cycle. We hypothesize that exposure to EE—via sensory, motor, psychological, and cognitive stimulation—may confer essential physiological benefits in females, including enhanced regulation of body weight and hormone secretion mediated by neuroendocrine axes. Ultimately, these effects may contribute to mitigating health-related impairments. Furthermore, we emphasize the importance of incorporating hormonal assessments—such as cortisol, corticosterone, and estradiol levels—alongside metabolic and inflammatory parameters. This integrated approach is essential to elucidate the mechanisms by which EE may enhance stress resilience at both cellular and systemic levels. Finally, we highlight that EE represents a cost-effective and highly feasible intervention. The standardization of EE protocols concerning animal density, experimental objectives, and exposure duration will be critical for advancing our understanding of its impact across various health and disease contexts.

## 6 Conclusion

Three weeks of environmental enrichment effectively reduced oxidative stress markers, enhanced enzymatic antioxidant activity, and increased levels of non-enzymatic antioxidant compounds in both hepatic and muscular tissues. Additionally, EE significantly decreased serum levels of IL-6, indicating a systemic anti-inflammatory effect in juvenile female mice.

## Data Availability

The raw data supporting the conclusions of this article will be made available by the authors, without undue reservation.
